# Recruitment effort and costs from a multi-center randomized controlled trial for treating depression in type 2 diabetes

**DOI:** 10.1186/s13063-019-3712-x

**Published:** 2019-11-06

**Authors:** Barbara A. Myers, Yegan Pillay, W. Guyton Hornsby, Jay Shubrook, Chandan Saha, Kieren J. Mather, Karen Fitzpatrick, Mary de Groot

**Affiliations:** 1 Indiana University School of Medicine, Diabetes Translational Research Center, 410 W 10th St, Suite 3100, Indianapolis, IN 46202 USA; 2 Patton College of Education, Ohio University, McCracken Hall 432M, Athens, OH 45701 USA; 3 West Virginia University School of Medicine, PO Box 9227, 8316 HSS, Morgantown, WV 26506 USA; 4 College of Osteopathic Medicine, Touro University California, 1310 Club Dr, Vallejo, CA 94592 USA

**Keywords:** Study recruitment, Diabetes, Depression, Clinical trial

## Abstract

**Background:**

Participant recruitment for clinical trials is a significant challenge for the scientific research community. Federal funding agencies have made continuation of funding of clinical trials contingent on meeting recruitment targets. It is incumbent on investigators to carefully set study recruitment timelines and resource needs to meet those goals as required under current funding mechanisms. This paper highlights the cost, labor, and barriers to recruitment for Program ACTVE II, a successful multisite randomized controlled trial of behavioral treatments for depression in adults with type 2 diabetes, conducted in rural and urban settings in three states.

**Methods:**

Quantitative and qualitative data on recruitment were gathered from study staff throughout the study recruitment period and were used to calculate costs and effort. The study utilized two main approaches to recruitment: (1) relying on potential participants to see ads in the community and call a toll-free number; and (2) direct phone calls to potential participants by study staff.

**Results:**

Contact was attempted with 18,925 people to obtain the enrolled sample of 140. The cost of recruitment activities during the 4.5-year recruitment period totaled $190,056, an average cost of $1358 per enrolled participant. Qualitative evaluations identified multiple barriers to recruitment.

**Conclusions:**

Recruitment for Program ACTIVE II exemplifies the magnitude of resources needed to reach recruitment targets in the current era. Continuous evaluation, flexibility, and adaptation are required on the part of investigators, community partners, and funding agencies to successfully reach high-risk populations in rural and urban areas.

**Trial registration:**

ClinicalTrials.gov, NCT03371940. Registered on 13 December 2017.

## Background

Randomized controlled trials (RCT) remain the gold standard for evaluating the effectiveness of interventions in healthcare research. Critical to the success of these trials is recruitment of study participants in order to have sufficient statistical power to detect the effects of interventions [1]. Recruitment is increasingly challenging for clinical trials across all domains of health research, particularly for studies recruiting from the community. Potential barriers include characteristics and stigma associated with the condition being studied, differences in cultural expectations and experience with research across study sites, and challenges engaging community healthcare providers and systems. However, because translational trials evaluate the effectiveness of interventions within the community [2], these studies are crucial to evaluating the implementation of these interventions in real-world contexts.

Mental health studies can be particularly challenging for recruitment. For those with major depressive disorder (MDD), withdrawal from regular activities is common. Eligible people may be less likely to engage and more difficult to retain in clinical trials, even if leaving the house to attend study appointments is not required [3]. A review of recruitment to depression treatment trials offering cognitive behavioral therapy (CBT) by computer reported enrollment rates of 2%–60% and uptake rates (actually participating in the treatment) of 3%–25%, representing the wide range of variability in acceptance of and participation in depression treatment trials [4]. The stigma of mental health conditions is an additional barrier for many [5]. Participating in mental health studies requires both acceptance of the condition and willingness to address it. This may create a significant struggle for the participant if shame or stigma are involved [6].

An individual’s culture, social background, and community also powerfully influence their perception of research and likelihood of participation [7, 8]. In many rural, medically underserved areas, patients commonly have a low opinion of the quality of their locally available healthcare and limited direct experience with participation in clinical research [9]. Research teams seen as an extension of this establishment may be viewed with skepticism [10]. In this setting, investigators need to build trust and perform recruitment activities simultaneously. This trust takes time to build and establish and relies on relationships among researchers, patients, and care providers. For example, the Appalachian region is well-known for its overrepresentation of a number of serious medical problems [11]. Despite the need, there are significant misgivings of the medical system among many people in this region [9]. This can lead to delays in care and resistance to enrolling in trials [12, 13].

Multicenter trials necessarily deal with these cultural values at each of the different study sites, with each community having its own sets of values. Efforts to build trust and creative solutions to study-related problems that work in one area may not work in another. This represents an added barrier to successfully recruiting and demands even more effort and flexibility among members of the research team.

The purpose of this paper is to present a case study of recruitment and retention for Program ACTIVE II, a successful, NIDDK-funded behavioral health trial [14]. We present our recruitment strategy, barriers, and costs associated with this trial.

## Methods

### Study design

Program ACTIVE II was a multisite RCT for adults with type 2 diabetes and MDD [14]. The purpose of the study was to test the comparative effectiveness of manualized (i.e. conducting an intervention according to a manual) exercise (EX) and cognitive behavioral (“talk”) therapies (CBT) for the treatment of depression, individually or in combination (EX+CBT), against usual care (UC). The study protocol was approved by the Institutional Review Boards at each of the individual sites (Indiana University 1105005684, 1308973934; Ohio University 11F031; West Virginia University H-23246).

Potential study participants were identified using a variety of methods, described below, from the communities surrounding the three participating study sites. Research staff trained in the study protocol and approved by the local site Institutional Review Boards (IRB) conducted all contacts with potential participants to assess their interest and eligibility. During screening calls, information was provided about the study using an approved script. If interested, participants gave verbal consent to continue with eligibility screening. Self-reported medical information was collected (see Table 1 for medical exclusion criteria). Those not excluded based on medical information were then screened for psychiatric eligibility. Potential participants who were eligible at medical and psychiatric screening by phone were invited to an in-person baseline eligibility assessment (baseline) where each potential participant provides written informed consent before completing any study-related activities. These baseline assessments were held at fitness centers within the three areas where recruitment took place. Each person then completed a psychiatric interview by phone. Following all baseline activities, an enrollment committee formally evaluated baseline results to determine eligibility and appropriateness for the study for each potential participant. Those not eligible were referred to other services. Eligible participants were randomized, notified of their randomization group (EX, CBT, EX+CBT, or UC), and assigned to an intervention provider, if relevant. Details of the study design and eligibility criteria have been detailed elsewhere [15].

**Table 1:** Study exclusion criteria

Medical exclusions	Psychiatric exclusions
• Age 18 years or older • Not able to walk • Currently pregnant or planning on becoming pregnant in the next year • History of diabetic ketoacidosis • Continuous insulin therapy since type 2 diabetes diagnosis • History of stroke or transient ischemic attack • Recent cardiac event (diagnosed angina, PTCA, any cardiac intervention for CAD or tachydysrhythmias in the past 6 months) • Laser surgery for proliferative retinopathy in the past 6 months • Aortic stenosis or other sever valvular heart disease or atrial fibrillation • Lower limb amputation • Asensory proliferative retinopathy • Uncontrolled hypertension • Current use of daytime oxygen (severe COPD) • Class III or IV heart failure • Diagnosis of type 2 diabetes < 1 year	• No diagnosis of current MDD • Active suicidal ideation or history of suicide attempt • Bipolar depression • Evidence of psychotic symptoms or history of psychotic disorder • Current substance abuse or dependence • Co-morbid anxiety or eating disorders where MDD is not the primary presentation • If on antidepressant medication, stable dose and usage for at least 6 weeks • Current therapy for MDD by a mental health professional

*PTCA* Percutaneous transluminal coronary angioplasty, *CAD* Coronary artery disease, *MDD* major depressive disorder, *COPD* chronic obstructive pulmonary disease

### Recruitment approach

Participants were recruited from rural southeastern Ohio (OH), north-central West Virginia (WV), and central Indiana (IN) communities. To ensure that recruitment goals were met, the study utilized multiple recruitments approaches, continually assessing the effectiveness of each, and adjusting strategies as necessary [16]. We classified recruitment identification strategies by who made the initial contact. Initial contacts initiated by the *potential participant* were classified as Inbound and those the *study team* initiated as Outbound.

#### Inbound recruitment strategies

Inbound recruitment used a flexible, multifaceted approach, involving outreach to physicians, health departments, libraries, grocery stores, pharmacies, advertisements in newspapers, and flyers posted in public areas. As recruitment methods were implemented, each was reviewed for effectiveness, altering or expanding as necessary to meet recruitment goals.

The main initial recruitment strategy for the study was via physician practices. This included providers affiliated with the study and those seeing patients with diabetes (e.g. endocrinologists, primary care providers, nurse practitioners). Providers were presented study information and asked if contact with patients could occur (e.g. letters to patients, in-office contact, phone calls to patients). Patients who received study information were instructed to call the study team. This outreach was later expanded to include pharmacists, diabetes educators, and diabetes education classes. Study staff also attended health fairs hosted by healthcare providers, community organizations, social justice groups, employer-hosted employee health fairs, and diabetes education groups, providing information about diabetes and depression, study promotional materials, and study flyers. Additional flyers and posters were distributed throughout the communities at libraries, churches, community centers, pharmacies, grocery stores, and to health departments to distribute at diabetes classes.

Advertisements were placed in local community newspapers. Limited advertising was also instituted on Facebook, targeting adult users with an interest in diabetes. Further outreach occurred via radio public service announcements (at the OH site), television segments (OH and WV), and radio segments (IN). These media spots aired locally on public radio and television stations as well as during local evening news broadcasts.

Additional outreach was conducted by direct mail or email to fitness facility members (at the IN and OH sites) and patient lists (WV and IN). Study emails were also sent to registrants of the Indiana Clinical and Translational Sciences Institute’s (CTSI) clinical trials registry (INResearch.org). At the IN site, advertisements were placed on the university’s electronic classifieds board, accessible by students, staff, and faculty.

#### Outbound recruitment strategies

Electronic medical records from physician practices and volunteer research registries were used to identify prospective participants, who were then directly contacted by the study team. This strategy was utilized at the IN and WV sites. WV conducted outreach to local physician offices. Interested practices allowed the study team contact patients first by mail and then by phone. The IN site partnered with ResNet, a service of the Indiana CTSI, to access patient data in the Eskenazi and IU Health systems. In addition, the IN site utilized the Indiana CTSI’s INresearch.org participant registry. After sending initial emails introducing the study, registrants with type 2 diabetes were contacted by a research assistant. The Ohio University IRB did not approve Outbound calling at the OH site. Unlike the other two sites, OH did not have institutional agreements with healthcare systems to share protected health information with researchers to facilitate Outbound outreach.

### Analyses

All contacts with potential participants were logged at the time of contact into a study database. Contact information included the date, time, and nature of the contact. Logged contacts included phone calls (such as study introduction calls, screening, scheduling, psychiatric interviews, etc.), mailings (such as appointment scheduling letters), and in-person appointments (such as eligibility assessments). In addition to logging the contact, the time needed to complete the contact was also recorded, allowing us to track the effort spent on various recruitment tasks. These records were reviewed to determine time spent on recruitment activities, broken down into phone screening and baseline eligibility assessment contacts. Descriptive statistics were used to characterize count, cost, and proportions of individuals in each outcome category. Qualitative data were collected from process notes recorded from team meetings over the study period.

## Results

### Quantitative data

#### Individuals screened and enrolled

**Fig. 1:**
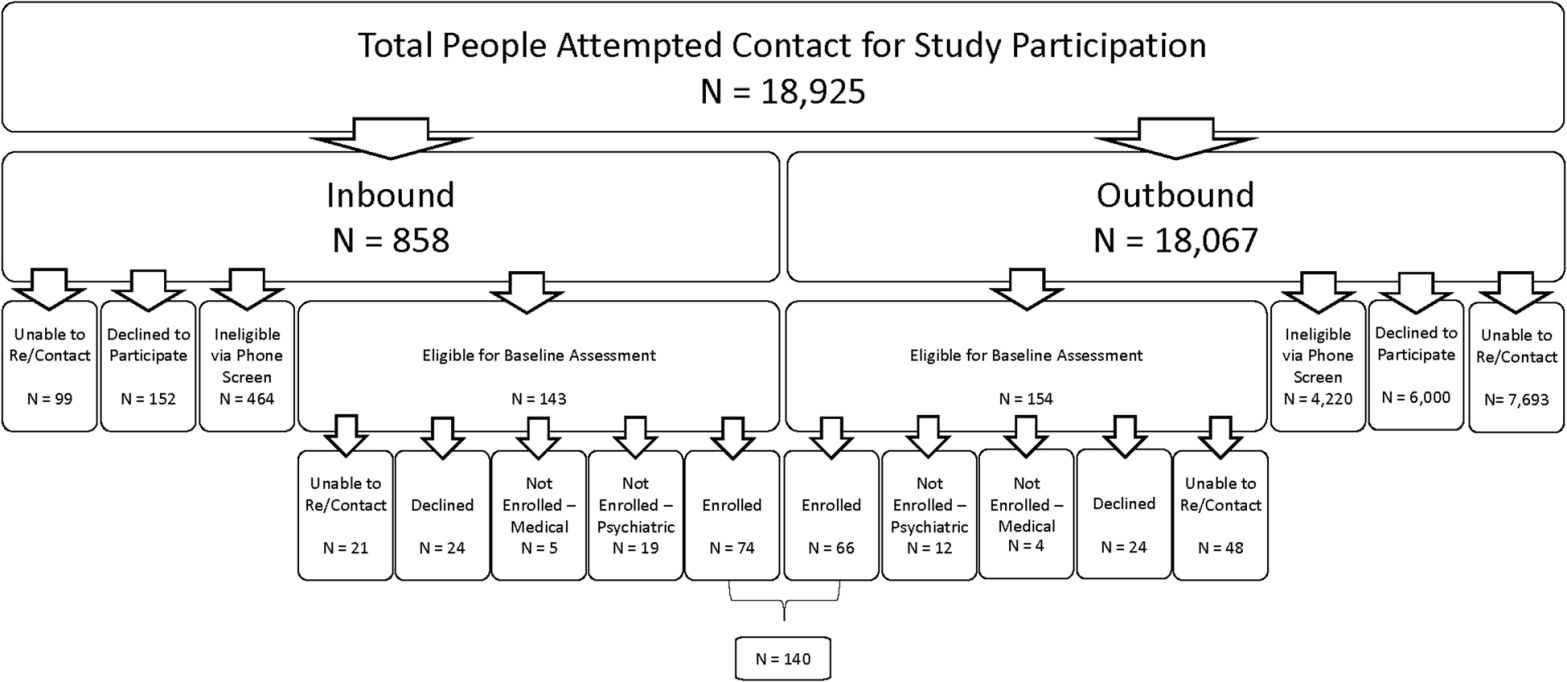
Enrollment flow chart: Inbound vs Outbound

Figure 1 characterizes recruitment flow using Inbound and Outbound strategies. A total of 18,925 potential participants were attempted contact, representing all patients on call lists for Outbound recruitment (*n* = 18,067) plus individuals who responded Inbound to community-based recruitment (*n* = 858) across the three study sites. For Inbound callers, 143 screened eligible for baseline while 464 were ineligible during phone screening. Of the 143 Inbound respondents who were referred for baseline, 74 enrolled in the study.

For Outbound recruitment, 154 people were eligible for baseline while 4220 were ineligible at phone screening. Another 6000 individuals declined participation and 7693 could not be reached for screening. Of the 154 respondents who were referred for baseline, 66 completed the assessment and enrolled in the study. The enrollment rate across the population in which contact was attempted was approximately 1% (140 enrolled divided by 18,925 people total).

Differences in recruitment rates were observed between the Inbound and Outbound approaches (Fig. 1 and Table 2). Forty-three percent of individuals could not be contacted from the Outbound calling list while 12% of Inbound respondents could not be re-contacted. Of those who were reached, the rate of self-withdrawal from consideration was three times higher in the Outbound approach compared with Inbound approach (58% vs 20%). For those who were interested and completed phone screening, ineligibility was frequent in both groups though notably higher in those contacted using the Outbound call strategy (96% vs 76%). Although the numbers of Outbound (*n* = 18,067) and Inbound (*n* = 858) pools of people vastly differed, similar numbers of individuals screened eligible for baseline (Outbound = 154 versus Inbound =143). Of those who were reached for phone screening, the success rate of ultimately enrolling the study participants was 15 times higher in the Inbound calling group compared with the Outbound calling group (9.7% vs 0.6%).

**Table 2:**
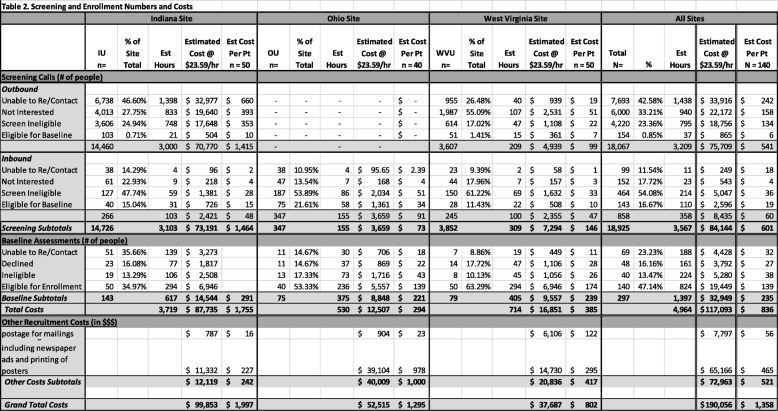
Screening and enrollment numbers and costs

**Table 3:** Study limitations and potential biases

Study limitations	Potential biases
• Staff effort may be underestimated due to inconsistent logging of time • Reported costs have not been adjusted for inflation	• Stringent study exclusion criteria increased recruitment effort and costs • Recruitment of people with MDD made recruitment more difficult • Some staff members were not consistent in logging time spent on tasks, resulting in some estimation of effort

#### Effort required to recruit and enroll participants

**Fig. 2:**
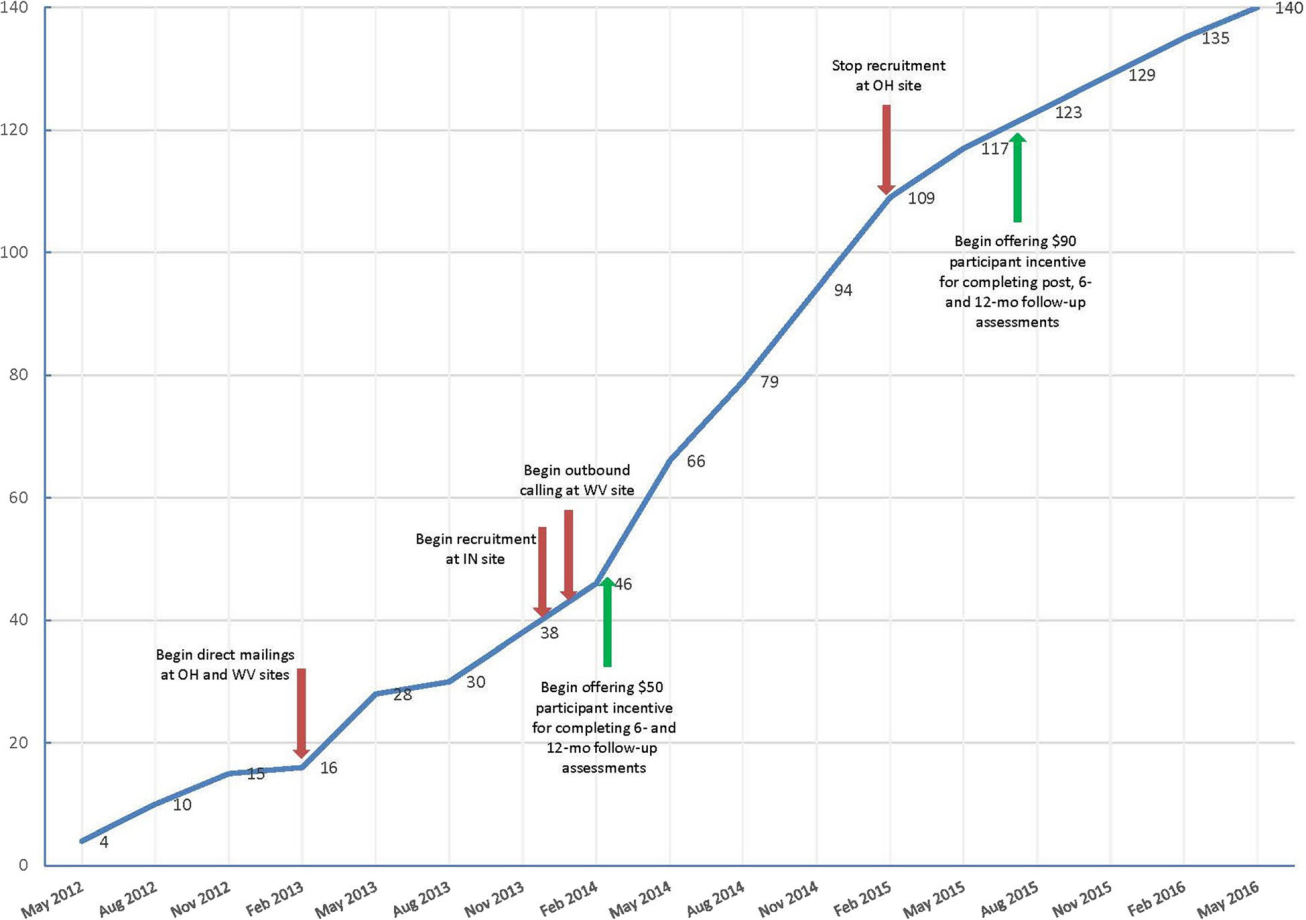
Cumulative participant enrollment

Recruitment efforts for the study began in May 2012 at the WV and OH sites (see Fig. 2). Based on data collected during the pilot study (R34DK71545) [17], we anticipated that it would be feasible to recruit the entire sample from the WV and OH sites. However, because recruitment rates at these two sites were lower than expected, recruitment began at the IN site in December 2013 to expedite enrollment. December 2013 also marked the start of Outbound calling at the WV site. As a result of these changes, the recruitment rate rose from 5.4 participants per quarter to 14.2 participants per quarter. This rate continued to February 2015 when recruitment ceased at the OH site. Because WV started recruitment without the use of Outbound calling, the enrollment rate there demonstrates the effectiveness of the Outbound strategy by increasing the average quarterly enrollment rate from 0.6 to 2.9 participants enrolled per quarter.

**Table 4:** Key points

• Recruitment to clinical trials is challenging • Recruitment planning during the grant application phase needs to reflect a realistic timeline and adequate budget • Recruitment efforts for Program ACTIVE II cost an average of $1358 for each of the 140 enrolled participants over four years • Sufficient time, funds, and funder support as well as flexibility and adaptation are necessary to meet recruitment goals	

Several key developments during the recruitment period are evident in Fig. 2. These included direct mailings by the OH and WV sites in February 2013, the initiation of Outbound recruitment strategies by the IN and WV sites in January 2014, cessation of recruitment efforts at the OH site in February 2015, and an increase in incentives to participants to enhance recruitment and retention in February 2014 and July 2015.

In order to estimate staff effort needed to achieve recruitment goals, average times for each contact activity were calculated. Group mean values were used to estimate for the missing entries. For all phone screening recruitment efforts, the estimated cumulative time spent on these efforts was 3567 h, the majority of which was consumed by Outbound calling (3209 h). An average of 25.5 h of personnel time was spent on all phone recruitment activities for each person enrolled (Table 2).

A total of 1397 h of staff time was spent on baseline activities for the 297 people who screened eligible by phone. This included time for scheduling assessments, mailing letters, in-person assessment activities, and the full psychiatric interview. Of the 297, 140 people completed baseline and were eligible for study participation. The average time for eligibility assessments to reach 140 participants was 10 h per enrolled participant. Adding the baseline time to the phone screening time, a total of 4964 h was required for all direct recruitment and enrollment efforts, an average of 35.5 h per participant enrolled, to obtain the final enrollment of 140 participants.

#### Costs of participant recruitment

Staff labor and advertising costs were calculated to determine the total cost of recruitment per participant. Staff labor rates were figured at $23.59 per hour to include both salary and benefits. The total calculation of all eligibility screening activities was $117,093 (see Table 2). Outbound calls accounted for the majority of recruitment expenditures ($75,709) due to the time involved. The remaining phone screening figure, $8435, was attributable to Inbound calls. Baseline activities added $32,949 in expenditures. The average amount to screen and enroll each participant into the study was $836.

Additional funds were used for advertising and outreach. Across all three sites, an estimated $72,963 was spent on newspaper advertising, printing posters, and postage for direct mailings. This figure is comparable to the Outbound calling efforts. Total sum of all recruitment, screening, and enrollment activities was estimated at $190,056, which represents an average of $1358 per participant enrolled in the study.

### Qualitative observations

The study team across all three sites met 1–2 times per month throughout the funding period. During these meetings, the study investigators discussed a variety of barriers, giving context to the extraordinary effort required by the study teams to meet recruitment goals. These barriers included geographic factors, institutional factors, study personnel factors, and cultural and social barriers, as we describe next.

#### Geographic factors

The differing geographical and cultural landscape at each of the study sites provided opportunities and challenges for recruitment. Physical distance and transportation infrastructure posed challenges to recruitment efforts at the rural OH and WV sites. The lack of public transportation in rural communities presented a unique problem to recruitment that could have been a factor in motivation to participate in the study. Participants were not compensated for travel to and from assessment sites and some locations for assessments were a fair distance from residences. Ohio and West Virginia participants lived an average of 11 miles and 13.5 miles from the closest assessment sites, respectively, which were located in communities surrounding the study sites in order to be closer to the population areas where the participants lived. However, some participants at these sites lived as far as 20–25 miles from the nearest assessment site, for a round trip as much as 50 miles away from the locations were the study procedure would take place. This present a significant burden to the participants, particularly those who were randomized to receive both CBT and exercise interventions, for as many as two appointments per week during the intervention period.

#### Institutional factors

The recruitment strategies were influenced by personnel composition and the research climate within each university. Ohio recruitment took place at Ohio University, which is predominantly a teaching institution. While Ohio University has ties to many of the 29 Appalachian counties in southern Ohio, the history and reach of clinical trials have traditionally been on a smaller scale. Formal collaborative partnership agreements with private practices and health systems in the surrounding area did not exist before the start of the study and needed to be established on an individual basis. A total of five healthcare systems partnered with OH over the course of the study. A request to the Ohio University IRB to make Outbound calls to potential participants was denied, limiting this site to the use of Inbound recruitment strategies.

West Virginia University has a long-standing history of clinical research, with the majority being conducted in the basic sciences and T1 translational trials. Institutional infrastructure leveraged for recruitment at this site included partnership with the West Virginia University CTSI, collaborative research agreements between the university and multiple healthcare systems serving northern WV, and a large-scale electronic medical record system from which eligible patients could be identified for recruitment.

In Indiana, recruitment was conducted at the Indiana University School of Medicine (IUSM), the lead study site. IUSM along with the Indiana CTSI have long-standing collaborations with the Eskenazi Health and Indiana University Health systems to collaborate on participant recruitment for clinical trials. These agreements, in partnership with the Regenstrief Institute’s Data Core, facilitated access to patient lists in order to conduct Outbound recruitment by identifying potentially eligible participants. Additional assistance was provided by the Indiana CTSI Research Network (ResNet), whose research assistants performed the majority of Outbound calling to medically screen interested patients and refer eligible patients to the study team.

Institutional differences across the sites expanded or limited the options available to investigators in reaching potential participants. While all sites were influenced by structural and regulatory changes in healthcare markets and delivery that occurred during the study period from 2012 to 2016, each site entered this period of change with different characteristics, resulting in more or less dramatic changes during the study period. For example, the communities around OH underwent a dramatic transformation in the consolidation of many small private primary care practices into larger healthcare organizations. Contractual contingencies on productivity expectations of physicians in these organization placed an implicit and, at times, an explicit limitation on physician efforts to convey study information to patients. While business managers and physicians acknowledged the benefits of how the study could complement their work and the potential benefits for patients, they were hindered by time and resource restrictions, which is consistent with the finding of other researchers in rural environments with limited resources [18].

#### Study personnel factors

The organization of the study teams contributed to relative strengths and challenges for each site. For example, at WV, study staffing was initially provided by graduate students. While this provided high-quality talent, the nature of the program limited the amount of time students could serve as study employees. This staff turnover contributed to challenges associated with establishing relationships with community organizations. A full-time project coordinator was hired in year 3, which facilitated greater continuity in building relationships with community partners as well as planning and implementation of new strategies. At OH, development of a new research center precipitated changes in study staffing, which ultimately precluded ongoing participation of OH in further enrollment.

#### Cultural and social barriers

Cultural barriers to recruitment included the burden of living with MDD, limited prior experience with clinical trials, and stigma associated with depression. Initial Inbound recruitment approaches needed potential participants to be sufficiently motivated to call a toll-free number. Since study inclusion criteria required the diagnosis for MDD, it is likely that some potential participants were unable to participate due to the manifest symptoms of depression such as hopelessness, lack of energy, anhedonia, and impaired social interactions. A significant number (296 people, 34.5%) who made the call to the toll-free number did not meet full criteria for MDD, which supports the hypothesis that people with higher burden of depression were less likely to contact the study team. Recruitment strategies such as mailings and fliers posted at clinics, doctor’s offices, churches, and community centers have a lower response rate when compared to recruitment strategies such as the use of a professional interviewer to follow up a mailing or word of mouth [19, 20].

The target communities at each study site differed in levels of experience with clinical trials. Individuals living in rural areas have limited exposure to clinical trials compared to their urban counterparts, which appeared to contribute to greater hesitancy to respond to Inbound recruitment efforts. Perceptions of researchers as outsiders and the perception of participation in research as being a “guinea pig” contributed to hesitancy to engage in screening.

The benefits of participating in a RCT may have been viewed with reluctance, especially given that participants could not be promised tangible benefits other than what they may receive by being randomly assigned to a treatment group. As a result, randomization to a specific treatment group without choice may have dissuaded individuals from volunteering. People who are seeking treatment for MDD frequently do not wish to be assigned to a Usual Care group, risking the possibility of receiving no treatment for the three months of the intervention period. This resulted in some eligible participants declining to participate in the study. Additionally, the possibility of being assigned to a counseling group may have influenced participation due to stigma associated with counseling.

At the IN site, clinical trials are more familiar to potential participants, but those trials typically involve fewer demands on participant time and engagement. Participants employed in hourly positions working in service industries consistently reported difficulty in knowing their work schedules far enough in advance to be able to keep study appointments. As a result, adaptations were made to accommodate work schedules. In addition, the target participant population at this site had an expectation of payment for study participation. A decision to institute and increase participant incentives across all sites was made on the basis of these public expectations.

## Discussion

Community recruitment of participants is critical to the advancement of all forms of science, particularly behavioral translational trials. Increasingly, clinical trials must compete for participant attention, interest, and time against a backdrop of considerable life demands and opportunities to easily obtain reliable health information. Funders and investigators recognize the central role that recruitment plays in the success of funded research. Underestimation of the effort and costs needed for successful recruitment is a modifiable variable that investigators can and should address during the study planning phase of the research. Here, the Program ACTIVE II study [14] experience is shared as a case example of the breadth and depth of recruitment strategies that were required to overcome barriers to study success as well as the monetary costs incurred (Table 3).

Endemic to the Program ACTIVE II study design, we encountered the following challenges: the specific eligibility criteria of the study (duration of type 2 diabetes for at least one year while meeting full DSM-IV-TR criteria for current MDD; medically appropriate for community-based exercise) [21], requirements for study participation (attending 2–3 appointments required for the baseline assessment, 10 weekly appointments for the CBT intervention, six exercise classes over eight weeks for the exercise intervention, and two appointments for each of three follow-up assessments) [21–23], stigma associated with both type 2 diabetes and depression [24], participant beliefs about study participation (e.g. lack of personal gain) [23, 24], diversification of communication channels to reach potential participants [22], health literacy issues [22], and co-morbid illnesses that may impede participation in studies [22, 23]. Each of these characteristics may have been relevant during study recruitment and reflected in the effort necessary for successful study enrollment.

These findings from the Program ACTIVE II recruitment experience demonstrate the considerable time, effort, and costs associated with recruiting participants for this federally funded behavioral RCT. These expenditures far exceeded investigator expectations and demonstrated important lessons for investigators and sponsors of future clinical trials. The costs incurred specifically for recruitment activities were nearly $1400 per participant, with $836 of this amount attributable to the direct costs required for recruitment of each enrolled participant, in line with direct recruitment costs reported by other diabetes studies [25, 26]. While grant application budgets tend to be focused on the costs associated with interventions, recruitment costs are substantial and should not be underestimated.

A second lesson was the amount of time needed to meet our recruitment target. Our team anticipated that we would complete recruitment activities within the first 24 months of the funding period. Instead, we required 4.5 years to achieve our goal. The extension of this period was a function of the large number of people we needed to contact to compete enrollment. The recruitment rates of this study (0.6%–1%) were significantly lower than those observed in our previous pilot study (8%) that used identical eligibility criteria [17], though similar to the 1.3% recruitment rate experienced by another depression treatment trial [27]. In the Program ACTIVE pilot work, Inbound recruitment approaches were successfully used to achieve a smaller target sample of 50 [17]. However, replication of those same methods did not yield similar recruitment rates in the larger trial at the same site (Table 4).

Continual reassessment of strategies and adaptations to the recruitment plan were needed throughout the recruitment period. The Outbound calling method was particularly important for Program ACTIVE II as people with depression may not seek mental health treatment because of stigma [5, 28, 29] or take the initiative to make the initial contact with a study [30]. This was especially relevant for our study considering that we recruited people with current MDD, who are relatively unlikely to initiate treatment for their depression [31].

There are limitations to the data presented in this paper. First, tracking of staff activities was not the primary goal of study data collection so some estimation was required in our calculations. As a result, the total effort of study staff may be underestimated for some activities (we erred on the side of underestimation). Additionally, figures reported are based on actual costs over the period of recruitment (2012–2016) and have not been adjusted for inflation. These numbers should be adjusted by investigators for use in future research budgets.

A critical element in the ultimate success of this trial was the flexibility and continued support by the funding agency as the investigators adapted to barriers to recruitment. The agency permitted additional time to complete recruitment and demonstrated confidence in the investigators, who were the most knowledgeable about their local barriers and needs. Had restrictions been placed on study funding based original assumptions about recruitment rates and strategies, the investigators would not have been able to demonstrate the effectiveness of the interventions [32].

## Conclusions

Recruitment remains the primary challenge to clinical trials. Innovation and flexibility in response to changes in the environment are critical to success. It is imperative that investigators plan conservatively for recruitment effort and costs during the grant application stage. Despite significant recruitment challenges, investigators should be mindful of meeting recruitment milestones or risk continued funding. The percentage of studies being funded has decreased substantially over the past 10 years [32], so it is imperative that those trials that are funded are successful in reaching their stated recruitment targets. However, not all conditions can be anticipated so investigators must be allowed flexibility as well. Policies that place study funding contingencies based upon achieving short-term recruitment targets should balance the risk of expenditures of failed studies against the loss of scientific knowledge and intervention development due to premature termination of studies requiring additional time and resources to meet recruitment goals.

## Data Availability

The datasets used and/or analyzed during the current study area available from the corresponding author on reasonable request.

## Abbreviations

CBT: Cognitive Behavioral Therapy, or “talk therapy,” intervention
EX: Exercise intervention
EX+CBT: Combined exercise and talk therapy intervention
IN: Indiana University Indianapolis study site
MDD: Major depressive disorder
OH: Ohio University study site
UC: Usual Care control
WV: West Virginia University study site
